# Monepantel (Veterinary Medicinal Products)

**DOI:** 10.14252/foodsafetyfscj.D-20-00001

**Published:** 2020-03-27

**Authors:** Food Safety Commission of Japan

## Abstract

FSCJ conducted a risk assessment of monepantel (CAS No.887148-69-8), a parasiticide based on results from various studies. Data on pharmacokinetics (cattle) and residues (cattle) were newly submitted. Negative results were obtained in all genotoxicity and carcinogenicity studies. The no-observed-adverse-effect level (NOAEL) obtained in all studies was 100 ppm (equivalent to 3 mg/kg bw per day for both sexes). In the 52-week chronic toxicity study in dogs, FSCJ specified an ADI for monepantel at 0.03 mg/kg bw per day based on NOAEL of 3 mg/kg bw per day, by applying a safety factor of 100.

## Conclusion in Brief^1^^)^

FSCJ conducted a risk assessment of monepantel (CAS No.887148-69-8), a parasiticide based on results from various studies. Data on pharmacokinetics (cattle) and residues (cattle) were newly submitted.

The data used in the assessment include pharmacokinetics (rats, dogs, sheep and cattle), residues (sheep and cattle), genotoxicity, acute toxicity (rats), subacute toxicity (mice, rats and dogs), chronic toxicity (rats and dogs), carcinogenicity (mice and rats), and reproductive and developmental toxicity (rats and rabbits) as well as data on general pharmacology.

Negative results were obtained in all genotoxicity studies. Therefore, it is possible to establish an acceptable daily intake (ADI) due to no genotoxicity relevant to humans. Monepantel did not show carcinogenicity in a 78-week carcinogenicity study in mice and a 104-week carcinogenicity study in rats.

Adverse effects observed at the lowest dose in various toxicological studies were shortened thromboplastin time, adrenal hypertrophy and histopathological changes in the liver of males, decreased albumin levels, decreased albumin/globulin ratio, increased alkaline phosphatase activity, and increased relative thyroid weight in females, at 300 ppm in a 52-week chronic toxicity study of dogs. The no-observed-adverse-effect level (NOAEL) in this study was 100 ppm (equivalent to 3 mg/kg bw per day for both sexes).

Consequently, FSCJ specified an ADI for monepantel at 0.03 mg/kg bw per day, based on NOAEL of 3 mg/kg bw per day in the 52-week chronic toxicity study in dogs, applying a safety factor of 100. (table 1)

**Table 1.** *Levels relevant to toxicological evaluation of monepantel*

Species	Study	Dose (mg/kg bw/day)	NOAEL (mg/kg bw/day)
Mouse	13-week subacute toxicity	M: 5, 18, 98, 959 F: 5, 22, 115, 1 213	5 Elevated AST levels
Mouse	78-week carcinogenicity study	1, 4, 16, 69	4 Increased incidence of fatty liver F: Increased absolute/relative liver weights Not carcinogenic
Rat	4-week subacute toxicity study	M: 86, 346, 1 044 F: 90, 362, 1 017	M: 86 (LOAEL) F: 90 (LOAEL) Centrilobular hypertrophy of hepatocytes M: Diffuse hypertrophy of thyroid follicular cells F: Increase in T.Chol, PL and TG as well as increased absolute/relative liver weight
Rat	90-day subacute toxicity	M: 4, 15, 74, 900 F: 4, 15, 81, 947	15 Increase in thromboplastin time F: Increase in T.Chol and PL, centrilobular hypertrophy of hepatocytes and increased absolute/relative liver weight
Rat	52-week chronic toxicity study	M: 3, 11, 54, 656 F: 3, 14, 67, 778	14 Increased absolute/relative liver weight
Rat	104-week carcinogenicity study	M: 5, 47, 578 F: 6, 57, 707	- Not carcinogenic
Rat	Two-generation reproductive toxicity study	F0: prior to mating: 15.8, 119, 950 During pregnancy: 13.5, 103, 863 During lactation period: 32.3, 245, 2 055 F_1_: prior to mating: 18.6, 141, 1 109 During pregnancy: 15.1, 114, 918 During lactation period: 30.8, 241, 2 028	Parent: 13.5 ~ 32.3 Increased absolute/relative liver weight, centrilobular hypertrophy of hepatocytes, cortical cell hypertrophy of the zona glomerulosa in the adrenal glands Offspring: 13.5 ~ 32.3 (LOAEL) Increased absolute/relative liver weight
Rat	Teratogenicity study	0, 100, 300, 1 000	1 000 Not teratogenic
Dog	4-week subacute toxicity	M: 161, 566, 1 217 F: 184, 561, 1 472	M: 161 (LOAEL), F: 184 (LOAEL) Increase in ALP, decreased absolute/relative thymus weight and increased absolute/relative adrenal weight
Dog	13-week subacute toxicity	M: 10, 107, 963 F: 11, 97, 1 176	(LOAEL) M: 10, F: 11 Small intestine (dilation of glands) F: Pancreas (increased apoptosis)
Dog	52-week chronic toxicity	M: 3, 10, 99 F: 3, 8, 91	3 M: Decrease in thromboplastin time and histopathological findings in the liver F: Decrease in Alb and A/G ratio, increase in ALP as well as increased relative thyroid weight
Rabbit	Teratogenicity	0, 100, 300, 1 000	1 000 Not teratogenic
Toxicological ADI (mg/kg bw/day)			0.03 NOAEL: 3 Safety factor: 100
The critical study for setting the ADI			52-week chronic toxicity study in dogs
ADI (mg/kg bw/day)			0.03
